# Ixekizumab-induced cutaneous vasculitis: A rare challenge in the era of biologic therapies

**DOI:** 10.1016/j.jdcr.2026.05.065

**Published:** 2026-06-04

**Authors:** Eine Yesid Benavides Tulcán, Sara Isabel Vanegas Arciniegas, Edgar Andrés Lozano Ponce

**Affiliations:** a Dermatology, Department of Internal Medicine, School of Health, Universidad del Valle, Cali, Colombia; b Dermatology, University Hospital of Valle, Cali, Colombia; c Department of Internal Medicine, Dermatology Section, Universidad del Valle, Cali, Colombia

**Keywords:** drug reaction, interleukin-17/antagonists and inhibitors, interleukin-23/antagonists and inhibitors, psoriasis, psoriatic arthritis, vasculitis

## Introduction

In recent years, anti-IL-17 biologics have demonstrated strong efficacy and favorable safety profiles in the treatment of psoriasis and psoriatic arthritis. Nevertheless, agents such as ixekizumab have been associated with specific adverse events, including mucocutaneous *Candida* infections and exacerbations of inflammatory bowel disease. In addition, a spectrum of *unexpected* and immune-mediated cutaneous reactions has been increasingly recognized.

Among these, vasculitis represents a particularly rare and poorly understood complication. Although only a limited number of cases have been reported in association with IL-17 inhibitors, these events raise important questions regarding the immunological mechanisms underlying vascular inflammation in the context of targeted biologic therapy. Recognizing such uncommon adverse reactions is essential for timely diagnosis, appropriate management, and therapeutic decision-making in patients receiving these agents.

## Case presentation

A 74-year-old woman with hypertension and a history of smoking was diagnosed with plaque and severe palmoplantar psoriasis. She presented with sharply demarcated erythematous scaly plaques with white borders on both soles, and with papules and smaller scaly plaques on the dorsal feet and distal legs.

She had been treated with adalimumab (40 mg subcutaneously every 2 weeks) for 8 years, achieving a stable PASI 90 response.^1^^,^^2^ Eventually, the patient relapsed with painful plantar lesions and new-onset psoriatic arthritis. This was interpreted as secondary loss of efficacy rather than a paradoxical flare given the recurrence of previously controlled symptoms and joint involvement.

In July 2022, treatment was switched to ixekizumab, administered according to the standard induction regimen (160 mg at week 0, followed by 80 mg every 2 weeks).

**Fig 1:**
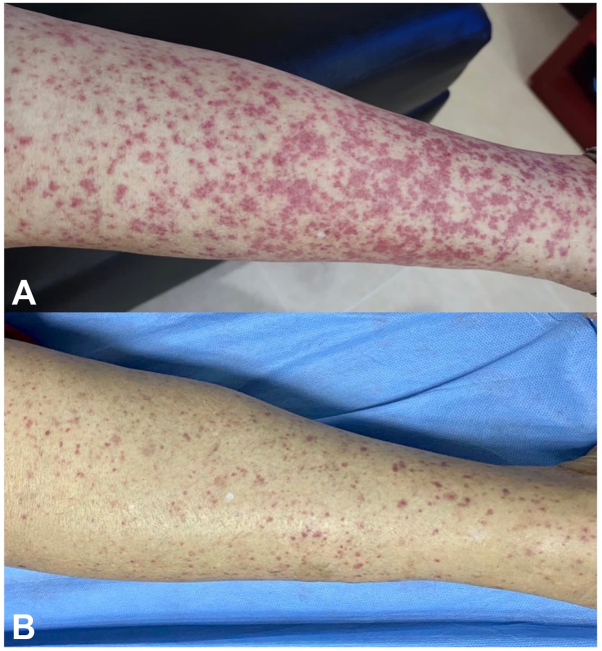
**A** and **B,** Well-defined purpuric papules and plaques on lower limbs.

After the second injection (week 2 of induction), the patient developed violaceous pinpoint macules and purpuric papules with a reticular distribution on the upper and lower extremities (Fig 1, *A*). She was evaluated in the emergency department and treated with oral prednisone (0.5 mg/kg/day, approximately 30 mg/day), with complete resolution of the lesions over the following 2 weeks. During this time the patient was not receiving ixekizumab.

In week 4, treatment with ixekizumab was continued. (With the third dose of the induction phase). Shortly after this third injection, the purpuric lesions recurred in a similar distribution (Fig 1, *B*). Given this recurrence upon re-exposure, ixekizumab was discontinued, and a skin biopsy was performed.

**Fig 2:**
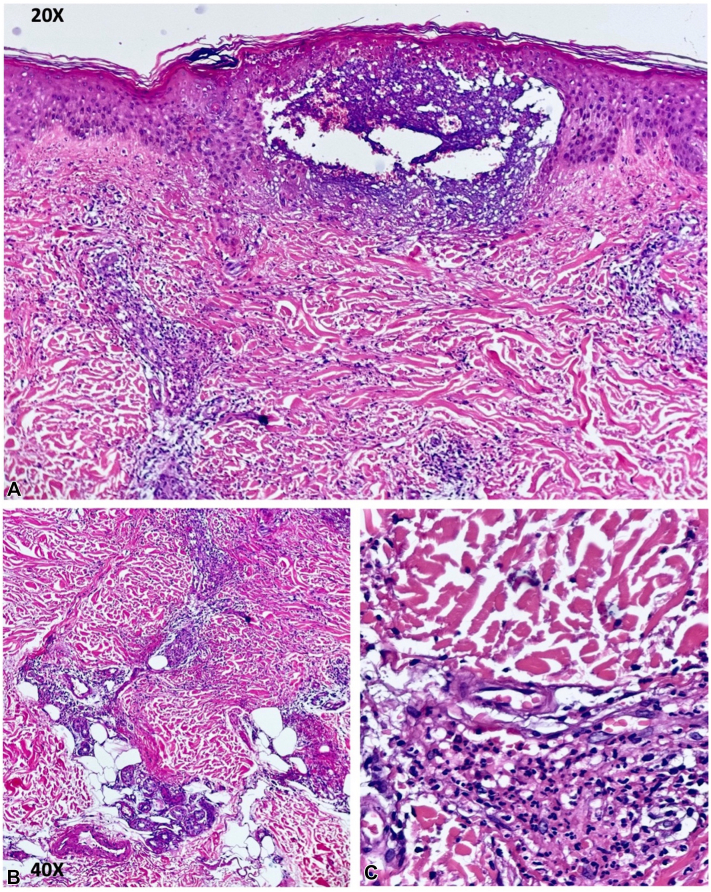
Hematoxylin and eosin (H&E) staining at different magnifications. **A,** Low-power view showing a superficial perivascular inflammatory infiltrate in the dermis. **B,** Intermediate magnification highlighting a dense neutrophilic infiltrate surrounding small dermal vessels. **C,** High-power view demonstrating leukocytoclasia with nuclear debris, fibrinoid necrosis of the vessel wall, and erythrocyte extravasation, consistent with leukocytoclastic vasculitis.

Histopathology revealed perivascular neutrophilic infiltrates with leukocytoclasia and fibrinoid necrosis, confirming leukocytoclastic vasculitis limited to the skin (Fig 2, *A*-*C*). Direct immunofluorescence (DIF) was not performed to further evaluate for IgA-associated vasculitis in the setting of leukocytoclastic vasculitis (LCV), due to its unavailability and the limited resources of our hospital. Extensive laboratory evaluation—including CBC, ESR, CRP, liver and renal function, autoimmune screening (ANA, ANCA, RF), cryoglobulins, hepatitis B and C serologies, syphilis serology, and urinalysis was unremarkable.

According to the Naranjo adverse drug reaction probability scale, a validated 10-item questionnaire designed to estimate the likelihood of a drug-related adverse event, the score was 7, indicating a probable drug-related event.The scale evaluates factors such as the temporal relationship between drug exposure and event onset, response to drug withdrawal, recurrence upon re-exposure, and exclusion of alternative etiologies.^3^, ^4^, ^5^ Other potential causes such as infection, concomitant medications (none newly introduced), or psoriasis-associated vasculitis were considered, but the clear temporal relationship, recurrence after rechallenging, and resolution upon withdrawal strongly implicated ixekizumab.

After multidisciplinary evaluation, therapy was switched to guselkumab (100 mg subcutaneously at weeks 0 and 4, then every 8 weeks). Guselkumab was selected for its demonstrated efficacy and safety in patients with immune-mediated conditions.^6^, ^7^, ^8^ Since the switch, the patient has remained free of vasculitic or other adverse events, and both her skin and joint symptoms have significantly improved, with only minimal residual plantar scaling.

This case raises important considerations regarding the immunologic pathways affected by IL-17 inhibition and the potential for paradoxical inflammation or autoimmunity.

## Discussion

Ixekizumab is a humanized monoclonal antibody targeting IL-17A, approved by the FDA and EMA for psoriasis, psoriatic arthritis, and spondyloarthropathies. Its efficacy and safety have been well established across clinical trials and real-world studies.^9^

However, *ixekizumab* adverse effects can occur. The most reported paradoxical 99 reaction is psoriasis itself, especially with TNFα inhibitors, but also observed with IL 17 blockers.^8^^,^^9^ This phenomenon may involve increased interferon-alpha production by plasmacytoid dendritic cells, with IL-17 playing a modulatory role.^10^ Other cutaneous side effects linked to IL-17 inhibition include eczematous dermatitis,^11^ possibly due to Th2 dominance and impaired antimicrobial peptide production by keratinocytes,^9^, ^10^, ^11^, ^12^ and neutrophilic dermatoses such as pyoderma gangrenosum, hidradenitis suppurativa, and Behçet disease, likely related to IL 23-driven neutrophilic inflammation.^13^

Rarely, vasculitis has been observed (Table I). Nine cases involving secukinumab have been published, including IgA vasculitis, granulomatosis with polyangiitis, and polyarteritis nodosa.^14^^,^^15^ For instance, a 56-year-old man developed systemic IgA vasculitis after secukinumab, with purpura, bloody diarrhea, nephropathy, and worsening psoriasis—possibly influenced by prior anti-TNFα exposure.^16^

**Table I:** Cutaneous vasculitis related with IL-17 inhibitors

Type of vasculitis	IL-17 inhibitor	Number of patient	Indications for us	Time to onset
Henoch-Schönlein purpura (HSP), now frequently termed IgA vasculitis	Secukinumab	1	Psoriasis	3 months
IgA vasculitis	Secukinumab	1	Anchylosis spondi	2 months
Cutaneous vasculitis	Secukinumab	2	Suppurative hidra	2 months
Leukocytoclastic vasculitis	Secukinumab	1	Psoriasis and psor	1 month
Segmental hyaline vasculitis	Secukinumab	1	Psoriasis	N/A
Granulomatosis with polyangiitis	Secukinumab	1	Psoriasis	N/A
Polyarteritis nodosa	Secukinumab	1	Psoriasis	N/A
Leukocytoclastic vasculitis	Ixekizumab	1	Psoriasis	N/A
Cutaneous small-vessel vasculitis	Brodalumab	1	Psoriasis	N/A

Mechanism	Management	Pathology	Reference
inmunocomplexes anti-IL-17	Drug withdrawal ± colchicine	Leucocytoclastic vasculitis	1
inmunocomplexes anti-IL-17	Drug withdrawal *n* + corticosteroids	Leucocytoclastic vasculitis	2
inmunocomplexes anti-IL-17	Withdrawal + topical corticosteroids	Leucocytoclastic vasculitis	3
inmunocomplexes anti-IL-17, cy	Drug withdrawal ± corticosteroids and colchicine	Fibrinoid necrosis in blood	4
N/A	N/A	Hyaline vasculitis	5
N/A	N/A	N/A	6
N/A	N/A	N/A	7
N/A	N/A	N/A	8
N/A	N/A	N/A	9

(1) Reverte M, Etienne M, Fouchard M, Doucet L, Brenaut E, Misery L. Occurrence of Henoch-Schön.

(2) Da Silva Cendon Duran C, Barreto Santiago M. Cutaneous Vasculitis During Secukinumab Treatm.

(3) Bostan E, Gulseren D, Yalici-Armagan B, Dogan S, Ates-Ozdemir D, Gokoz O, Kalyoncu U, Atakan.

(4) Chelli C, Loget J, Vanhaecke C, Durlach A, Gagneux-Lemoussu L, Soriano C, Viguier M. Cutaneous.

(5) ClinicalTrials.gov [Internet]. Bethesda (MD): NationalLibrary of Medicine (US). 2000. Feb 29. Iden.

(6) ClinicalTrials.gov [Internet]. Bethesda (MD): NationalLibrary of Medicine (US); 2000. Feb 29. Iden.

(7) ClinicalTrials.gov [Internet]. Bethesda (MD): NationalLibrary of Medicine (US). 2000. Identifier N.

(8) ClinicalTrials.gov [Internet]. Bethesda (MD): NationalLibrary of Medicine (US). 2000. Identifier NC.

(9) ClinicalTrials.gov [Internet]. Bethesda (MD): National Library of Medicine (US). 2000. Identifier N.

To date, only 1 case of leukocytoclastic vasculitis linked to ixekizumab has been reported,^17^ during the UNCOVER-3 trial, without detailed clinical description. Integrated safety analyses of over 20,000 patient-years of exposure have not identified vasculitis as a recurring signal. This case, therefore, is the second documented and the first published in a post-marketing setting.^14^, ^15^, ^16^, ^17^

The exact mechanism by which IL-17 inhibition may trigger vasculitis is unknown. Hypotheses include disruption of the Th17-neutrophil axis, immune complex deposition, or altered vascular homeostasis.^7^

## Conclusion

A variety of disorders have been associated with anti-IL-17 therapies such as ixekizumab, though most are rare, and causality is often unclear. Dermatologists must be aware of these potential adverse events—including rare cases of cutaneous vasculitis—and consider alternative treatments when appropriate.

## Conflicts of interest

None disclosed.
